# Relationship between Cigarette Smoking and Cancer Characteristics and Survival among Breast Cancer Patients

**DOI:** 10.3390/ijerph19074084

**Published:** 2022-03-30

**Authors:** Sarah Darmon, Amie Park, Leann A. Lovejoy, Craig D. Shriver, Kangmin Zhu, Rachel E. Ellsworth

**Affiliations:** 1 Murtha Cancer Center/Research Program, Uniformed Services University of the Health Sciences and Walter Reed National Military Medical Center, Bethesda, MD 20889, USA; sarah.darmon.ctr@usuhs.edu (S.D.); amie.park.ctr@usuhs.edu (A.P.); craig.shriver@usuhs.edu (C.D.S.); kangmin.zhu@usuhs.edu (K.Z.); 2 Henry M. Jackson Foundation for the Advancement of Military Medicine, Bethesda, MD 20817, USA; 3 Chan Soon-Shiong Institute of Molecular Medicine at Windber, Windber, PA 15963, USA; l.lovejoy@wriwindber.org; 4 Department of Surgery, Uniformed Services University of the Health Sciences, Bethesda, MD 20889, USA; 5 Department of Preventative Medicine and Biostatistics, Uniformed Services University of the Health Sciences, Bethesda, MD 20889, USA

**Keywords:** cigarette smoking, breast cancer, pathology, survival

## Abstract

Carcinogenic effects of tobacco smoke may affect breast tumorigenesis. To assess whether cigarette smoking is associated with breast cancer characteristics, we investigated the relationships between smoking, pathological characteristics, and outcomes in 2153 women diagnosed with breast cancer 2001–2016. Patients were classified as never, former, or current smokers at the time of diagnosis. Logistic regression and multivariable Cox proportional hazards analysis were performed to determine whether smoking was associated with tumor characteristics. Multivariable Cox proportional hazards analysis was conducted to compare former or current smokers to never smokers in survival with adjustment for the potential confounders. The majority of women (61.8%) never smoked, followed by former smokers (26.2%) and current smokers (12.0%). After adjustment for demographic variables, body mass index, and comorbidities, tumor characteristics were not significantly associated with smoking status or pack-years smoked. Ten-year overall survival was significantly lower for former and current smokers compared to never smokers (*p* = 0.0105). However, breast cancer specific survival did not differ significantly between groups (*p* = 0.1606). Although cigarette smoking did not alter the underlying biology of breast tumors or breast cancer-specific survival, overall survival was significantly worse in smokers, highlighting the importance of smoking cessation in the recently diagnosed breast cancer patient.

## 1. Introduction

Cigarette smoking is the leading cause of preventable morbidity and mortality in the US, with approximately 480,000 deaths in the US each year attributable to cigarette smoking (Health Effects of Cigarette Smoking|CDC; accessed on 29 September 2021). Cigarette smoking has been associated with a number of health conditions, including cardiovascular and pulmonary disease, cancer, adverse reproductive outcomes, and other chronic health conditions [1]. Smoking-related illnesses are estimated to cost the United States $225 billion in direct medical costs and $156 billion in lost productivity each year (Economic Trends in Tobacco|Smoking and Tobacco Use|CDC, accessed on 29 September 2021). In 2019, there were an estimated 34.1 million cigarette smokers in the United States (US), with 15.3% of adult males and 12.7% of adult females being current smokers [2].

While a number of studies suggest that smoking is a moderate risk factor for breast cancer, whether smoking influences tumor characteristics or survival is less clear. Identification of tumor phenotypes enriched in smokers may evince the underlying effects on tumor development. For example, the enrichment of *TP53* mutations in breast tumors from smokers [3] may promote the basal-like subtype, which is characterized by high rates (80%) of *TP53* mutations [4], while cigarette smoking has been reported to have anti-estrogenic effects [5], which may reduce the risk of estrogen receptor (ER) positive tumors. In conjunction, nicotine, which promotes the proliferation of human breast cancer cell lines [6], may result in larger tumor sizes in smokers compared to non-smokers. Thus, in this study, we investigated whether smoking status was associated with tumor characteristics and survival among female breast cancer patients.

## 2. Materials and Methods

Patients were enrolled in the Clinical Breast Care Project (CBCP) from breast clinics at the Murtha Cancer Center/Walter Reed National Military Medical Center (MCC/WRNMMC), Bethesda, MD, Anne Arundel Medical Center (AAMC), Annapolis, MD or Joyce Murtha Cancer Center (JMBCC), Windber, PA. Enrollment criteria included being an adult over the age of 18 years, mentally competent and willing to provide informed consent. Patients were provided with consent forms that included permission to gather demographic, pathological, and survival data and that described the primary research uses of the samples. All patients included in this study were diagnosed with invasive breast cancer between 2001 and 2016.

All data were extracted from the CBCP database. Smoking status was based on patient-reported past and current cigarette smoking. Smokers were defined as those who had smoked at least 100 cigarettes in their lifetime before breast cancer diagnosis. Former smokers were those who quit smoking ≥1 year prior to diagnosis. Pack-years were calculated as the product of the number of years smoked and number of packs smoked per day. Comorbidities were included in the analyses using the Charlson comorbidity index (CCI) calculated before breast cancer diagnosis. Additional demographic data included age at diagnosis, self-described race/ethnicity, body mass index (BMI), education levels, marital status, and site of patient enrollment. Pathological data included histological type, anatomic tumor stage, size, and lymph node status [7], and grade [8,9]. Biomarkers included in the analyses included estrogen receptor (ER), progesterone receptor (PR), and HER2, with positivity assigned according to ASCO/CAP guidelines [10,11]. Information on vital status and date at death was collected through annual review of electronic health records.

We first described the distributions of demographic characteristics, site of enrollment, BMI, and CCI between never smokers, former smokers, and current smokers using Chi-square test between never, former, and current smokers. We then compared the groups by smoking status in pathologic variables (type, stage, size, grade, ER status, PR status, HER2 status, and node status of tumor). Subsequently, we compared 25–49 or ≥50 pack-years to pack-years <25 in pathologic features. In these analyses, multivariable logistic regression analysis was conducted for each of the pathologic variables controlling for demographic variables, site of enrollment, BMI, and CCI. Patients with unknown demographic or pathological data were excluded. In survival analysis, we used Kaplan–Meier curve to compare the groups divided by smoking status in 10-year overall or breast cancer-specific survival. If a patient died during the 10-year period after diagnosis, the follow up time was calculated as the time from diagnosis to death. If death was not observed during the period, follow-up time was censored at the end of the tenth year. Patients who were not dead through the end of the study without a full ten-year follow-up time were censored on the study ending 31 December 2020. We then used multivariable Cox proportional hazards model to estimate hazard ratios (HRs) and 95% confidence intervals (CIs) for former and current smokers compared to never-smokers. In the Cox models, we adjusted for age, race, marital status, education, tumor type, tumor stage, tumor size, tumor grade, node status, tumor hormone receptor statuses (HER2, ER and PR), BMI, CCI, and site of enrollment. The same approach was used for the comparison of survival between the groups defined by pack-year.

## 3. Results

### 3.1. Study Characteristics

Between 2001 and 2016, 2153 women with invasive breast cancer enrolled in the CBCP had complete smoking information, 34 women were excluded for not having sufficient data to determine smoking status at the time of diagnosis. The majority of women (*n* = 1330, 61.8%) never smoked, followed by former smokers (*n* = 564, 26.2%) and current smokers (*n* = 259, 12.0%). Age at diagnosis, race/ethnicity, education levels, CCI and site of enrollment differed significantly by smoking status (Table 1). The average ages at diagnosis were 57.4 years, 61.2 years, and 55.5 years in never, former, and current smokers, respectively. Former smokers were less likely to be Non-Hispanic Black (NHB) or Asian or to have a college education and were more likely to have comorbidities than never smokers. Current smokers were less likely to be NHB, to have a college education or be married than never smokers.

### 3.2. Tumor Pathology

Tumor stage, size, and lymph node status differed significantly by smoking status, with the former smokers being more likely to have stage I, tumor size T1, and negative lymph nodes (Table 2). After multivariable analyses, adjusting for age at diagnosis, race, marital status, education levels, site of enrollment, BMI and CCI only lymph node status was associated with smoking status, with former smokers having lower risk (OR 0.76, 95% CI, 0.60–0.97) compared to never smokers (Table 3). Evaluation of the data by pack-year also found no significant differences in tumor characteristics between women who smoked 25–49 years or ≥50 years and those who smoked <25 years (data not shown).

### 3.3. Survival

**Figure 1:**
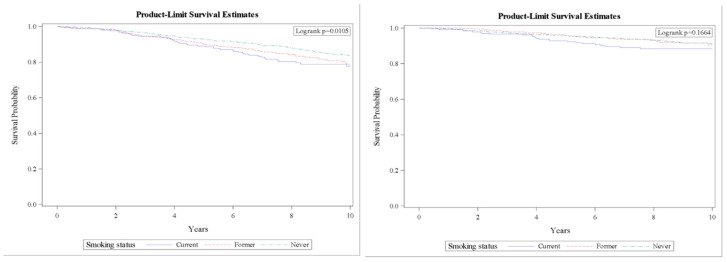
10-year survival curves for overall (**left**) and breast cancer specific survival (**right**). Controlled for age, race, BMI, CCI, marital status, education, treatment site, stage, tumor characteristics (grade, type, size), node status, and hormone receptor (HER2, ER, and PR).

BCSS did not differ significantly by smoking status (Figure 1) after adjustment for age, race, marital status, education, tumor stage, tumor characteristics (grade, type, size), node status, tumor hormone receptor statuses (HER2, ER, and PR), BMI, CCI, and site of enrollment, the HRs were 1.40 (95% CI, 1.04–1.88) and 1.59 (95% CI, 1.06–2.37) for former and current smokers for overall survival, and 1.44 (95% CI, 0.92–2.24) and 1.37 (95% CI, 0.79–2.40) for former and current smokers for BCSS, compared with non-smokers. Further analysis shows no significant differences in either overall survival or BCSS by pack-year: HRs were 0.97 (95% CI, 0.59–1.60) and 1.18 (95% CI, 0.67–2.08) for women with 25–49 and ≥50 pack-years for overall survival, compared with women who smoked <25 pack-years. The corresponding HRs were 0.89 (95% CI, 0.42–1.90) and 1.03 (95% CI, 0.40–2.67) for BCSS.

## 4. Discussion

Nicotine and its metabolites have been detected in breast fluids from nonlactating smokers, and many of the carcinogens in tobacco smoke cause mammary tumors in animal models [12,13], suggesting that smoking-derived carcinogens may be present in the breast and increase the risk of breast cancer. Data from this study suggest that while current smokers have worse overall survival, cigarette smoking is not associated with an enrichment of any tumor characteristics or breast cancer-specific survival.

Differences in tumor stage, size, and lymph node status, which reflect the time at which a tumor is resected, may be attributable not to smoking-derived carcinogen exposure in the breast but rather to other factors, such as decreased adherence to mammographic screening. In a study of 89,058 women enrolled in the Women’s Health Initiative, current smokers were significantly less likely to undergo mammographic screening with concomitant higher stage breast cancer diagnoses [14]. In contrast, former smokers were significantly more likely to undergo mammography. This higher use of regular screening may explain the lower rates of metastatic lymph nodes in former smokers detected in our study.

The association of smoking with breast cancer biomarkers has been mixed. In our study, no significant association was detected between smoking status and ER, PR, or HER2 status. Similarly, ER status did not differ significantly by smoking status in studies from the Cancer Prevention Study II study [15] or Multiethnic Cohort [16]. Data from the EPIC study, however, showed an association between current smoking and ER+ tumors (HR = 1.23, 95% CI 1.04–1.45) [17]. When evaluating tumor subtypes, smoking >20 years was associated with luminal (OR 1.51, 95% CI 1.19–1.93) but not basal-like (OR 0.90 95% CI 0.57–1.43) breast cancer [18]. In contrast, evaluation of smoking and subtype using data from the Surveillance, Epidemiology, and End Results (SEER) database found no difference in breast cancer subtypes by smoking status [19]. Despite these mixed results, these data suggest that the hypothesized antiestrogenic effects of cigarette smoking do not reduce the risk of ER+ tumors.

As with tumor characteristics, the association between cigarette smoking and BCSS is controversial. In our study, smoking status was associated with overall survival, but not BCSS. While some studies have found no association between smoking and BCSS [20,21,22,23,24], a number of other studies have found significant associations [25,26,27,28,29,30,31]. The study from Braithwaite et al. included a similar number of patients as our study (2265 and 2153, receptively) [27]. The Braithwhite et al. study, however, found a two-fold higher BCSS rate in current smokers compared to never smokers (HR = 2.01, 95% CI 1.27–3.18), while our study did not detect any differences in BCSS in current compared to never smokers (HR = 1.37; 95% CI, 0.79–2.40). This difference may be attributable to the length of follow-up. The average length of follow-up in the study by Braithwhite et al. was 12.3 years (11.9 years, 12.2 years, and 12.4 years for current, former, and never smokers). The average length of follow-up in our study was 8.1 years (7.42 years, 9.33 years, and 8.32 years for current, former, and never smokers, respectively). Additional studies that found an association between BCSS and current smoking had larger sample sizes and follow-up times of >10 years [29,30,31]. Data from the Carolina Breast Cancer Study evaluated BCSS in 1808 women and did not detect an association between smoking and BCSS at five years, however, 13-year conditional BCSS was elevated amongst current smokers [32]. Meta-analysis found that while all-cause mortality was significantly associated with smoking across all time periods, smoking was associated with BCSS only for those with follow-up >10 years [26]. Thus, we do not exclude the possibility that there is a long-term effect of smoking on breast cancer-specific survival if such effects occur after 10 years. In contrast, overall survival, despite adjusting for comorbidities, was significantly lower for both former and current compared to never smokers. These results suggest that smoking has general, rather than breast-specific, effects on health status.

In addition to follow-up <10 years, this study has limitations. For example, this study was a hospital rather than a population-based study. This cohort may not, therefore, be reflective of the general US population. In addition, our study did evaluate smoking status at diagnosis and did not include data on smoking cessation after diagnosis. Smoking cessation at or around the time of diagnosis has been recognized as a critical component in increasing the survival of lung cancer patients [33]. Smoking cessation has also demonstrated improved survival in breast cancer patients, with women who quit smoking at diagnosis having a 33% lower risk of death than those who continue to smoke [34,35,36]. The lack of significant differences in BCSS by smoking status in our study, therefore, may be impacted by the rate of smoking cessation. Finally, data regarding exposure to second-hand smoke was not collected in the CBCP questionnaires. In a study of 1755 women, Strumylaite et al. found that exposure to passive smoke was associated with an increased risk of breast cancer [37]. Risk increased with longer exposure, however, no significant association was detected for hormone receptor status. A second study 1508 women found no association between either overall or breast cancer-specific survival and exposure to second-hand smoke [38]. Thus, although the burden of breast cancer attributable to exposure to passive smoking could not be addressed in our study, previous studies do not suggest passive smoking is associated with breast cancer characteristics or survival

## 5. Conclusions

Cigarette smoking did not significantly alter tumor pathological characteristics of breast tumors. Although breast cancer-specific survival did not differ significantly, overall survival was significantly worse in smokers, and smoking cessation should be encouraged in the recently diagnosed breast cancer patient.

## Figures and Tables

**Table 1:** Demographic characteristics of 2153 women with breast cancer enrolled in the CBCP 2001–2016. *p*-values reflect differences between never, former, and current smokers.

Characteristic	Never (*n* = 1330)		Former (*n* = 564)		Current (*n* = 259)		*p*-Value
	N	%	N	%	N	%	
Age at Diagnosis							<0.0001
<45	243	18.3	50	8.9	38	14.7	
45–54	359	27.0	126	22.3	96	37.1	
55–64	340	25.6	165	29.3	75	29.0	
65–74	253	19.0	152	27.0	39	15.1	
75+	135	10.2	71	12.6	11	4.3	
Race/Ethnicity							0.0005 ^b^
NHW	997	75.0	470	83.3	216	83.4	
NHB	252	19.0	77	13.7	28	10.8	
Asian	43	3.2	7	1.2	7	2.7	
Hispanic	23	1.7	9	1.6	6	2.3	
Other/Unknown	15	1.1	1	0.2	2	0.8	
Education							<0.0001 ^b^
Less than 4 Year College ^a^	617	46.4	333	59.0	182	70.3	
4 Year College or Beyond	578	43.5	209	37.1	58	22.4	
Unknown	135	10.2	22	3.9	19	7.3	
Marital Status							0.2223 ^b^
Not Married	389	29.3	184	32.6	92	35.5	
Married	932	70.1	377	66.8	165	63.7	
Unknown	9	0.7	3	0.5	2	0.8	
BMI							0.1654 ^c^
Less than 25	430	32.3	158	28.0	97	37.5	
25–29.9	410	30.8	191	33.9	79	30.5	
30 or Greater	487	36.6	213	37.8	83	32.1	
Unknown	3	0.23	2	0.35	0	0.0	
CCI							<0.0001
0	370	27.8	99	17.6	67	25.9	
1	312	23.5	121	21.5	77	29.7	
2	291	21.9	147	26.1	59	22.8	
3+	357	26.8	196	34.8	55	21.2	
Unknown	0	0.0	1	0.2	1	0.4	
Site of enrollment							<0.0001
AAMC	627	47.1	361	64.0	140	54.1	
JMBCC	175	13.2	60	10.6	62	23.9	
WR	528	39.7	143	25.4	57	22.0	

^a^ Includes vocational or trade school; ^b^ Fisher’s exact test used instead of chi-square test due to small cell size less than 5; ^c^ Fisher’s exact test could not be computed, not enough resources; chi-square test used instead.

**Table 2:** Clinicopathological characteristics for 2153 women with breast cancer enrolled in the CBCP 2001–2016.

Characteristic	Never (*n* = 1330)		Former (*n* = 564)		Current (*n* = 259)		*p*-Value
	N	%	N	%	N	%	
AJCC Pathological Stage							0.0027
Stage I	684	51.4	340	60.3	118	45.6	
Stage II	446	33.5	161	28.5	93	35.9	
Stage III/IV ^a^	180	13.6	53	9.4	43	16.6	
Unknown	20	1.5	10	1.8	5	1.9	
Tumor Type							0.9031
IDCA	1013	76.2	432	76.6	202	78.0	
ILCA	157	11.8	61	10.8	29	11.2	
Other	140	10.5	65	11.5	26	10.0	
Unknown	20	1.5	6	1.1	2	0.8	
Tumor Grade							0.3289
Well Differentiated	331	24.9	149	26.4	52	20.1	
Moderately Differentiated	502	37.7	204	36.2	114	44.0	
Poorly Differentiated	450	33.8	191	33.9	87	33.6	
Unknown	47	3.5	20	3.6	6	2.3	
Tumor Size							0.0185
T1	836	62.9	382	67.7	143	55.2	
T2	377	28.4	145	25.7	87	33.6	
T3/T4	82	6.2	23	4.1	17	6.6	
Unknown	35	2.6	14	2.5	12	4.6	
ER Status							0.0530 ^b^
Negative	236	17.7	86	15.3	55	21.2	
Positive	1091	82.0	478	84.8	202	78.0	
Unknown	3	0.2	0	0.0	2	0.8	
PR Status							0.0972 ^b^
Negative	420	31.6	158	28.0	87	33.6	
Positive	905	68.1	406	72.0	170	65.6	
Unknown	5	0.4	0	0.0	2	0.8	
HER2 Status							0.4435
Negative	1114	83.8	476	84.4	206	79.5	
Positive	193	14.5	79	14.0	46	17.8	
Unknown	23	1.7	9	1.6	7	2.7	
Node Status							0.0005
Negative	853	64.1	409	72.5	153	59.1	
Positive	445	33.5	141	25.0	101	39.0	
Unknown	32	2.4	14	2.5	5	1.9	

^a^ To comply with HIPAA guidelines for presenting data in cells with N < 11, stage III and IV have been combined here. The *p*-value presented here was calculated with Stage III and IV as separate factors. ^b^ Fisher’s exact test was used instead of chi-square test due to small cell size of less than 5.

**Table 3:** Logistic regression model adjusted for age, race, marital status, education levels, site of enrollment, BMI, and CCI for women diagnosed with invasive breast cancer 2001–2016.

Adjusted OR for Smoker vs. Never Smoked			
Response Variable		Smoking Status	
		Current Smoker	Former Smoker
Tumor stage	Stage I	Reference	Reference
	Stage II	1.247 (0.895, 1.739)	0.795 (0.624, 1.012)
	Stage III	1.260 (0.771, 2.059)	0.832 (0.565, 1.224)
	Stage IV	2.675 (0.998, 7.170)	0.278 (0.063, 1.233)
Tumor type	IDCA	Reference	Reference
	ILCA	1.055 (0.654, 1.701)	0.876 (0.623, 1.233)
	Other	0.969 (0.580, 1.619)	1.002 (0.698, 1.438)
Tumor grade	Well-differentiated (grade 1)	Reference	Reference
	Moderately differentiated (grade 2)	1.460 (0.971, 2.193)	0.890 (0.675, 1.172)
	Poorly differentiated (grade 3)	1.207 (0.784, 1.857)	1.031 (0.775, 1.371)
Tumor size	T1	Reference	Reference
	T2	1.337 (0.963, 1.856)	0.957 (0.748, 1.224)
	T3/4	1.386 (0.755, 2.545)	0.755 (0.443, 1.287)
ER	Negative	Reference	Reference
	Positive	0.777 (0.527, 1.147)	1.031 (0.756, 1.406)
PR	Negative	Reference	Reference
	Positive	0.889 (0.643, 1.230)	1.166 (0.914, 1.488)
HER2	Negative	Reference	Reference
	Positive	1.209 (0.807, 1.812)	0.940 (0.680, 1.299)
Node	Negative	Reference	Reference
	Positive	1.235 (0.904, 1.688)	0.759 (0.595, 0.967)

## Data Availability

Data provided upon request.
